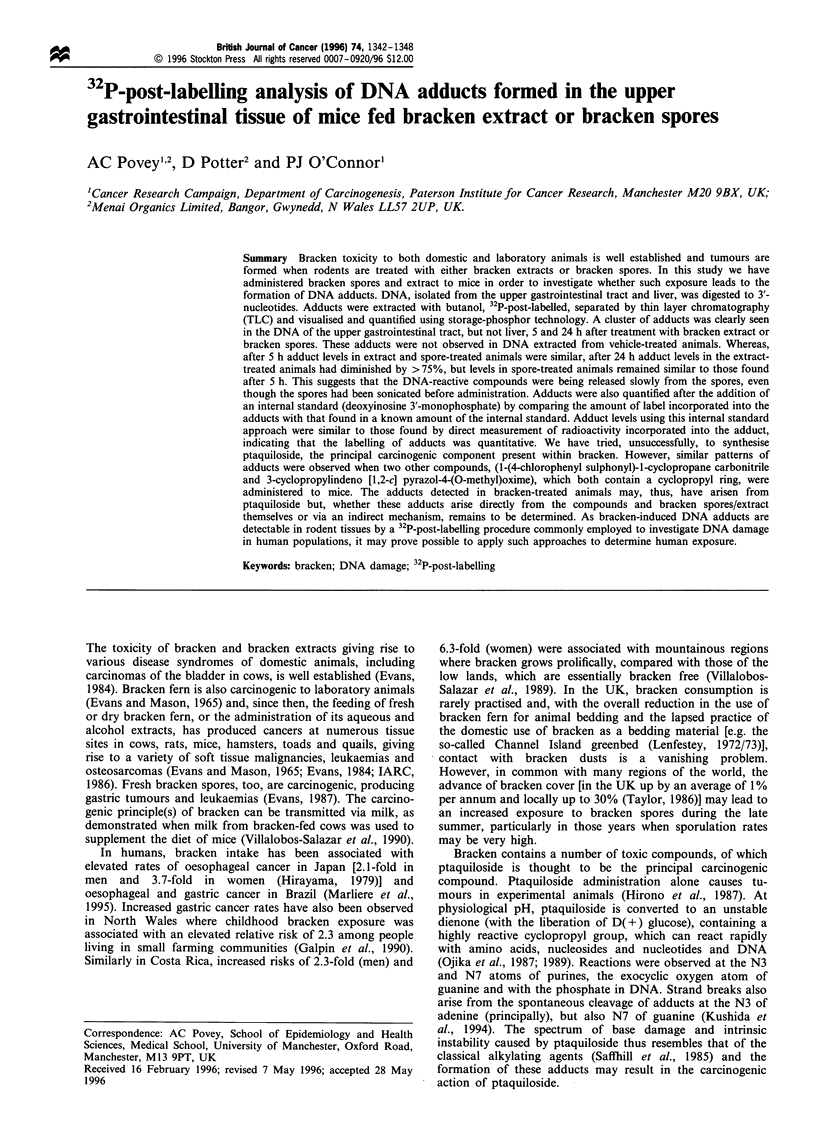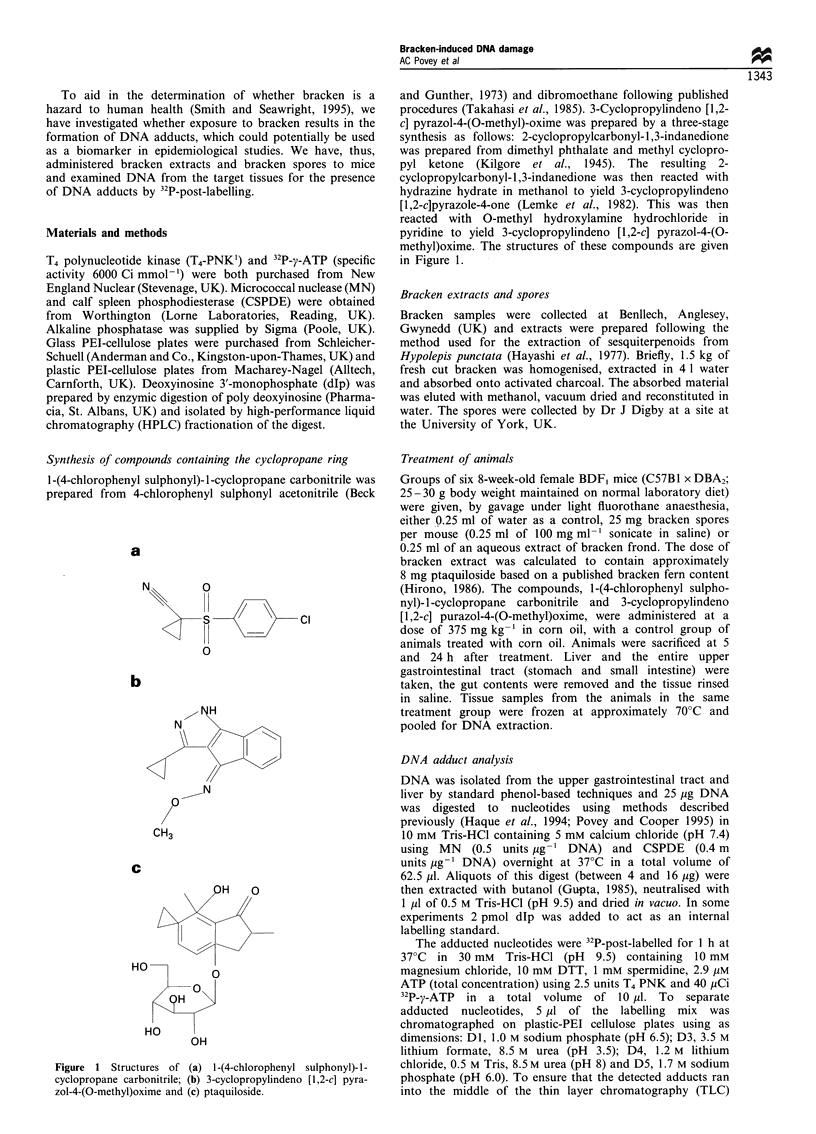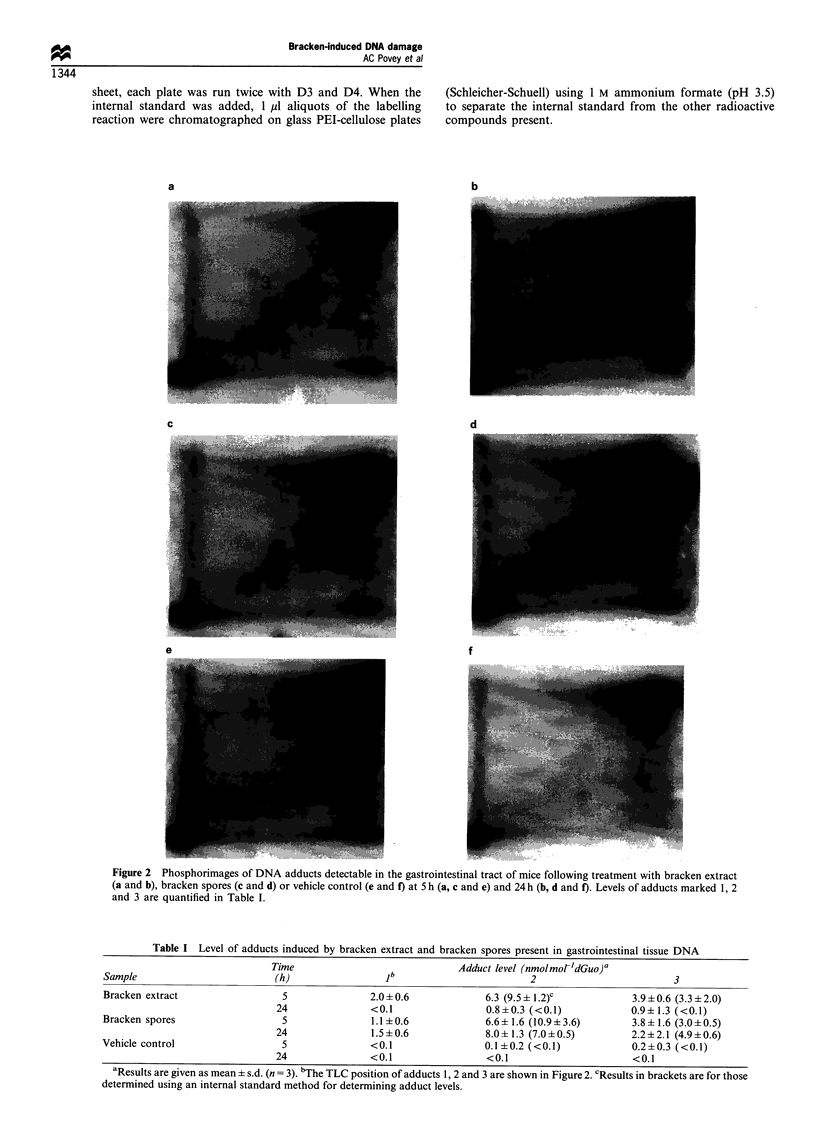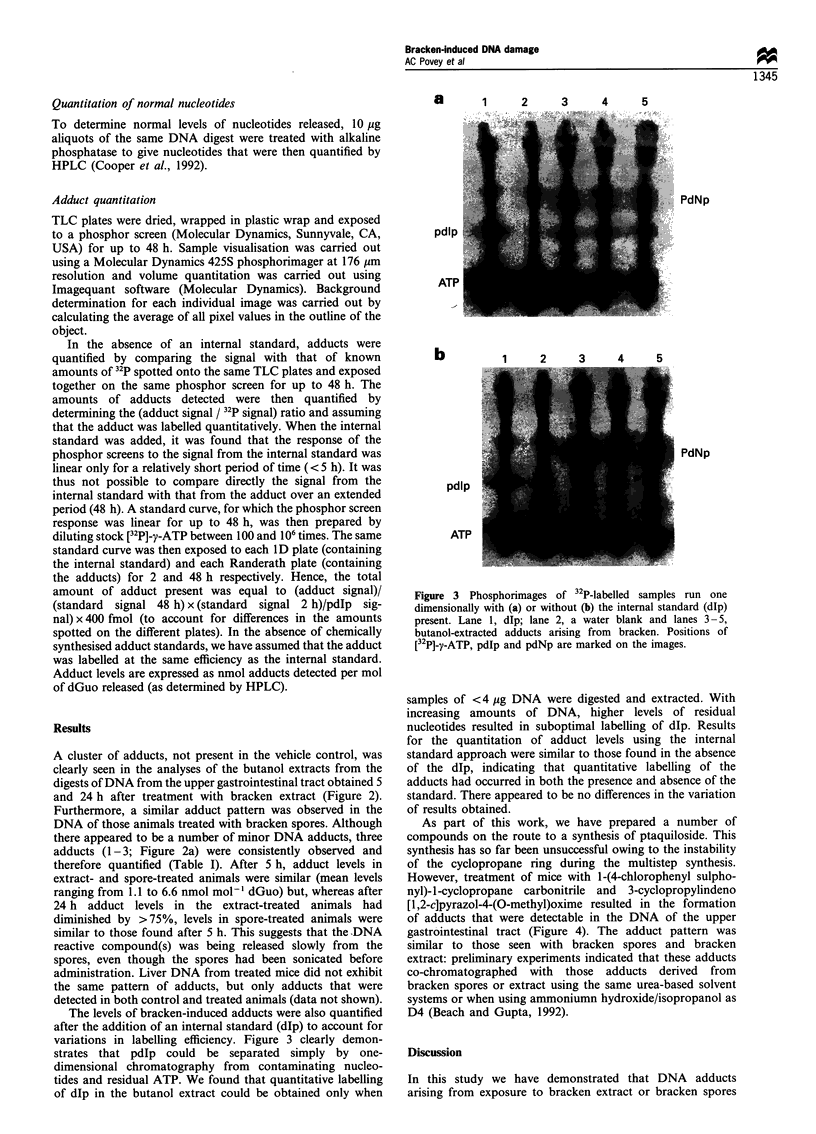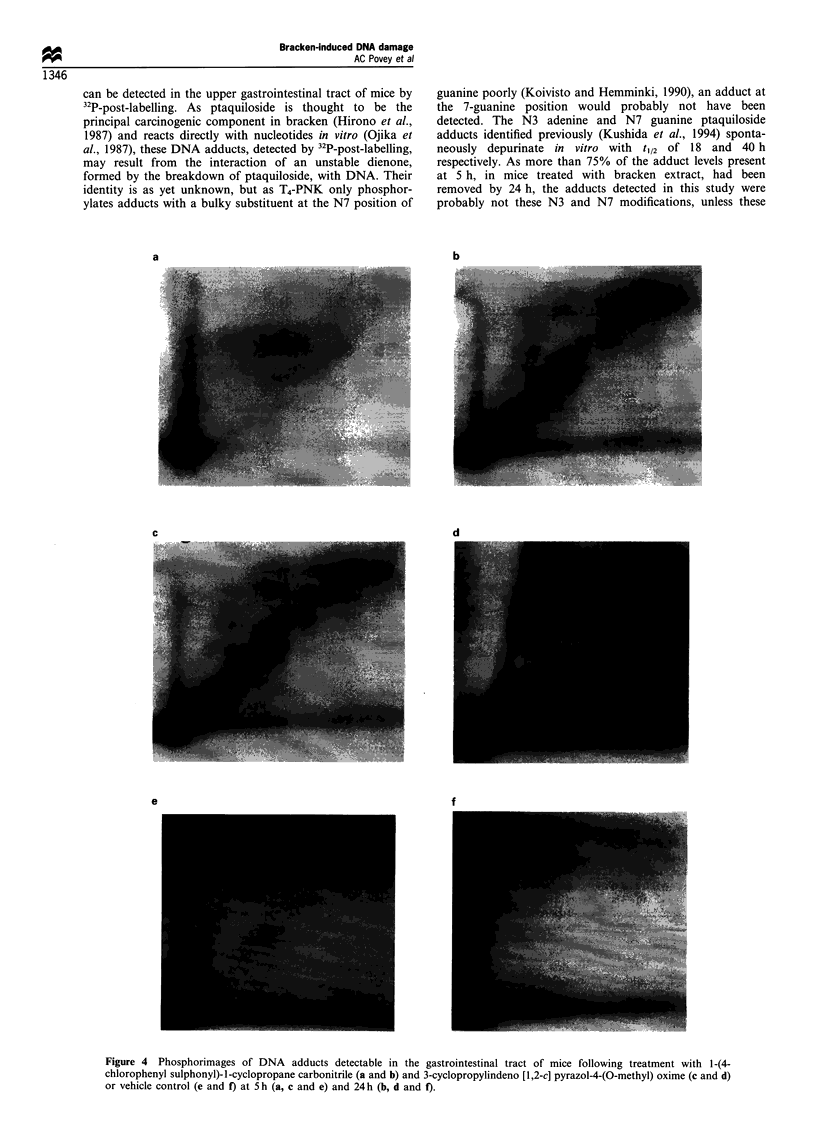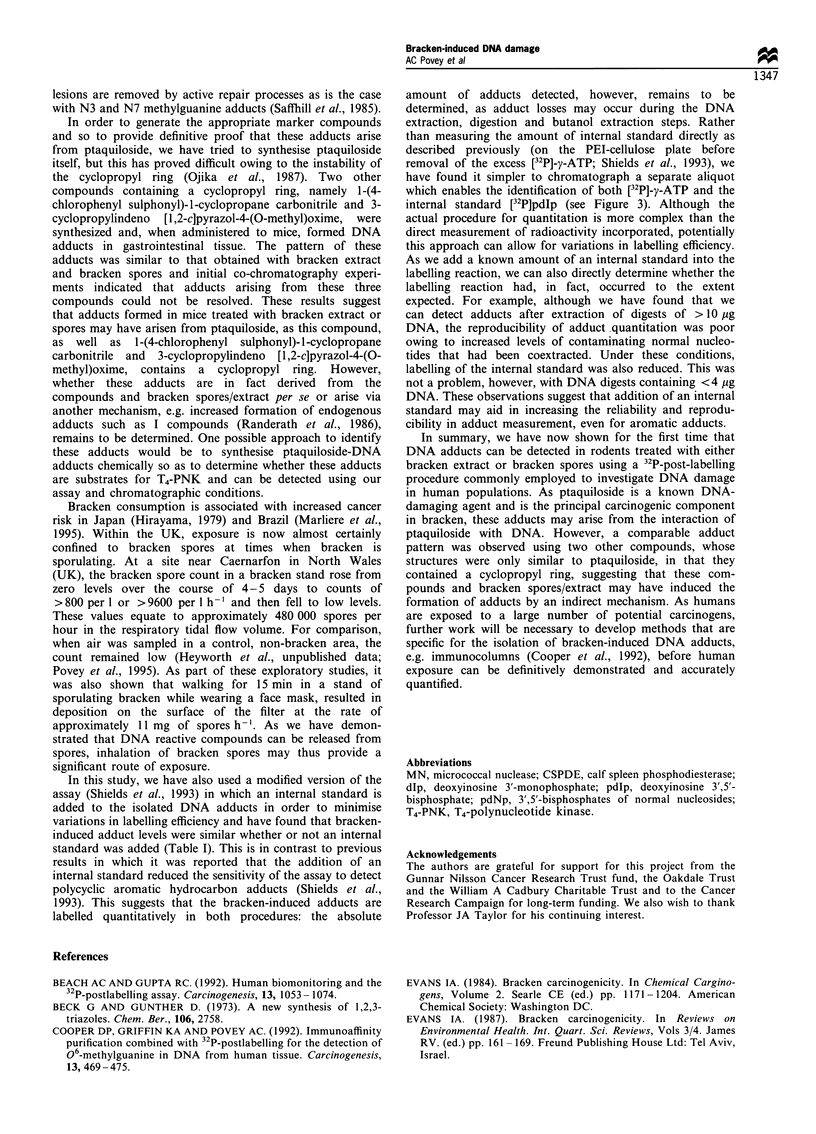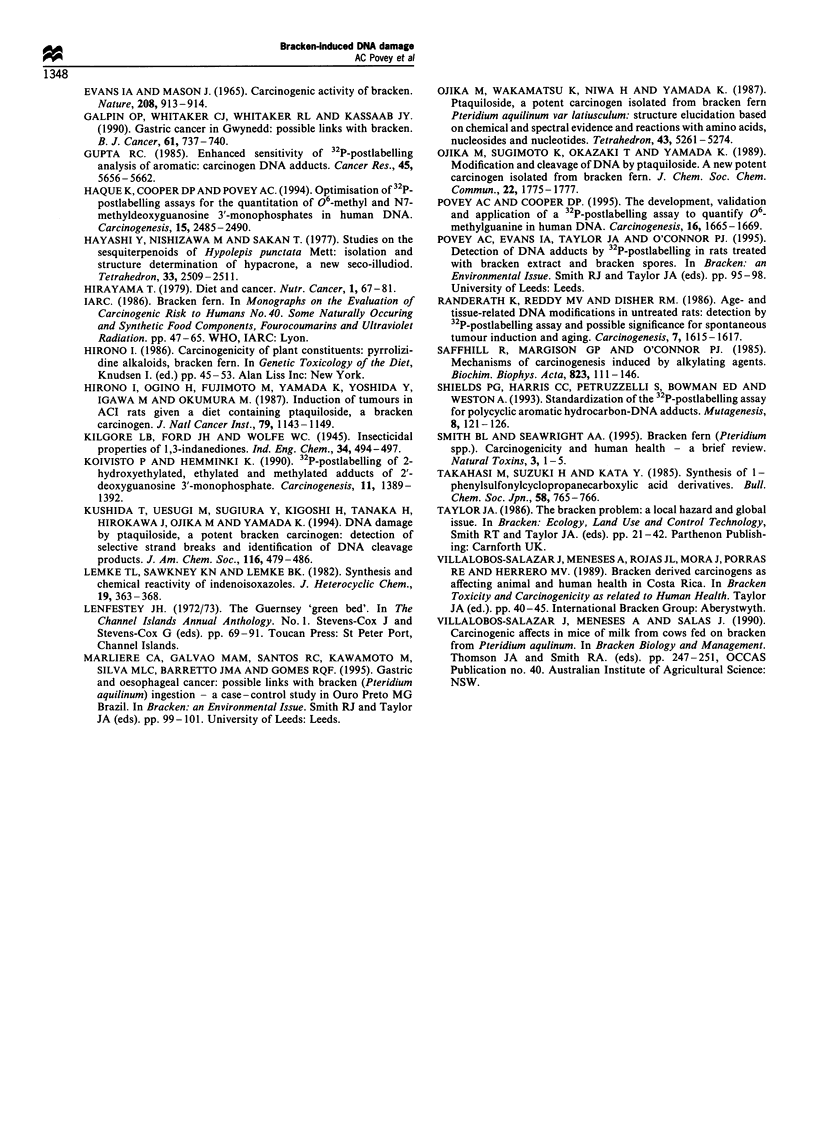# 32P-post-labelling analysis of DNA adducts formed in the upper gastrointestinal tissue of mice fed bracken extract or bracken spores.

**DOI:** 10.1038/bjc.1996.547

**Published:** 1996-11

**Authors:** A. C. Povey, D. Potter, P. J. O'Connor

**Affiliations:** Department of Carcinogenesis, Paterson Institute for Cancer Research, Manchester, UK.

## Abstract

**Images:**


British Journal of Cancer (1996) 74, 1342–1348
© 1996 Stockton Press All rights reserved 0007-0920/96 $12.00

### $^{32}$P-post-labelling analysis of DNA adducts formed in the upper gastrointestinal tissue of mice fed bracken extract or bracken spores

AC Povey[1,2], D Potter[2] and PJ O'Connor[1]

[1]*Cancer Research Campaign, Department of Carcinogenesis, Paterson Institute for Cancer Research, Manchester M20 9BX, UK;* [2]*Menai Organics Limited, Bangor, Gwynedd, N Wales LL57 2UP, UK.*

**Summary** Bracken toxicity to both domestic and laboratory animals is well established and tumours are formed when rodents are treated with either bracken extracts or bracken spores. In this study we have administered bracken spores and extract to mice in order to investigate whether such exposure leads to the formation of DNA adducts. DNA, isolated from the upper gastrointestinal tract and liver, was digested to 3′-nucleotides. Adducts were extracted with butanol, $^{32}$P-post-labelled, separated by thin layer chromatography (TLC) and visualised and quantified using storage-phosphor technology. A cluster of adducts was clearly seen in the DNA of the upper gastrointestinal tract, but not liver, 5 and 24 h after treatment with bracken extract or bracken spores. These adducts were not observed in DNA extracted from vehicle-treated animals. Whereas, after 5 h adduct levels in extract and spore-treated animals were similar, after 24 h adduct levels in the extract-treated animals had diminished by >75%, but levels in spore-treated animals remained similar to those found after 5 h. This suggests that the DNA-reactive compounds were being released slowly from the spores, even though the spores had been sonicated before administration. Adducts were also quantified after the addition of an internal standard (deoxyinosine 3′-monophosphate) by comparing the amount of label incorporated into the adducts with that found in a known amount of the internal standard. Adduct levels using this internal standard approach were similar to those found by direct measurement of radioactivity incorporated into the adduct, indicating that the labelling of adducts was quantitative. We have tried, unsuccessfully, to synthesise ptaquiloside, the principal carcinogenic component present within bracken. However, similar patterns of adducts were observed when two other compounds, (1-(4-chlorophenyl sulphonyl)-1-cyclopropane carbonitrile and 3-cyclopropylindeno [1,2-*c*] pyrazol-4-(O-methyl)oxime), which both contain a cyclopropyl ring, were administered to mice. The adducts detected in bracken-treated animals may, thus, have arisen from ptaquiloside but, whether these adducts arise directly from the compounds and bracken spores/extract themselves or via an indirect mechanism, remains to be determined. As bracken-induced DNA adducts are detectable in rodent tissues by a $^{32}$P-post-labelling procedure commonly employed to investigate DNA damage in human populations, it may prove possible to apply such approaches to determine human exposure.

**Keywords:** bracken; DNA damage; $^{32}$P-post-labelling

The toxicity of bracken and bracken extracts giving rise to various disease syndromes of domestic animals, including carcinomas of the bladder in cows, is well established (Evans, 1984). Bracken fern is also carcinogenic to laboratory animals (Evans and Mason, 1965) and, since then, the feeding of fresh or dry bracken fern, or the administration of its aqueous and alcohol extracts, has produced cancers at numerous tissue sites in cows, rats, mice, hamsters, toads and quails, giving rise to a variety of soft tissue malignancies, leukaemias and osteosarcomas (Evans and Mason, 1965; Evans, 1984; IARC, 1986). Fresh bracken spores, too, are carcinogenic, producing gastric tumours and leukaemias (Evans, 1987). The carcinogenic principle(s) of bracken can be transmitted via milk, as demonstrated when milk from bracken-fed cows was used to supplement the diet of mice (Villalobos-Salazar *et al.*, 1990).

In humans, bracken intake has been associated with elevated rates of oesophageal cancer in Japan [2.1-fold in men and 3.7-fold in women (Hirayama, 1979)] and oesophageal and gastric cancer in Brazil (Marliere *et al.*, 1995). Increased gastric cancer rates have also been observed in North Wales where childhood bracken exposure was associated with an elevated relative risk of 2.3 among people living in small farming communities (Galpin *et al.*, 1990). Similarly in Costa Rica, increased risks of 2.3-fold (men) and 6.3-fold (women) were associated with mountainous regions where bracken grows prolifically, compared with those of the low lands, which are essentially bracken free (Villalobos-Salazar *et al.*, 1989). In the UK, bracken consumption is rarely practised and, with the overall reduction in the use of bracken fern for animal bedding and the lapsed practice of the domestic use of bracken as a bedding material [e.g. the so-called Channel Island greenbed (Lenfestey, 1972/73)], contact with bracken dusts is a vanishing problem. However, in common with many regions of the world, the advance of bracken cover [in the UK up by an average of 1% per annum and locally up to 30% (Taylor, 1986)] may lead to an increased exposure to bracken spores during the late summer, particularly in those years when sporulation rates may be very high.

Bracken contains a number of toxic compounds, of which ptaquiloside is thought to be the principal carcinogenic compound. Ptaquiloside administration alone causes tumours in experimental animals (Hirono *et al.*, 1987). At physiological pH, ptaquiloside is converted to an unstable dienone (with the liberation of D(+) glucose), containing a highly reactive cyclopropyl group, which can react rapidly with amino acids, nucleosides and nucleotides and DNA (Ojika *et al.*, 1987; 1989). Reactions were observed at the N3 and N7 atoms of purines, the exocyclic oxygen atom of guanine and with the phosphate in DNA. Strand breaks also arise from the spontaneous cleavage of adducts at the N3 of adenine (principally), but also N7 of guanine (Kushida *et al.*, 1994). The spectrum of base damage and intrinsic instability caused by ptaquiloside thus resembles that of the classical alkylating agents (Saffhill *et al.*, 1985) and the formation of these adducts may result in the carcinogenic action of ptaquiloside.

Correspondence: AC Povey, School of Epidemiology and Health Sciences, Medical School, University of Manchester, Oxford Road, Manchester, M13 9PT, UK
Received 16 February 1996; revised 7 May 1996; accepted 28 May 1996

To aid in the determination of whether bracken is a hazard to human health (Smith and Seawright, 1995), we have investigated whether exposure to bracken results in the formation of DNA adducts, which could potentially be used as a biomarker in epidemiological studies. We have, thus, administered bracken extracts and bracken spores to mice and examined DNA from the target tissues for the presence of DNA adducts by $^{32}P$-post-labelling.

### Materials and methods

$T_4$ polynucleotide kinase ($T_4$-PNK[1]) and $^{32}P$-$\gamma$-ATP (specific activity 6000 Ci $mmol^{-1}$) were both purchased from New England Nuclear (Stevenage, UK). Micrococcal nuclease (MN) and calf spleen phosphodiesterase (CSPDE) were obtained from Worthington (Lorne Laboratories, Reading, UK). Alkaline phosphatase was supplied by Sigma (Poole, UK). Glass PEI-cellulose plates were purchased from Schleicher-Schuell (Anderman and Co., Kingston-upon-Thames, UK) and plastic PEI-cellulose plates from Macharey-Nagel (Alltech, Carnforth, UK). Deoxyinosine 3′-monophosphate (dIp) was prepared by enzymic digestion of poly deoxyinosine (Pharmacia, St. Albans, UK) and isolated by high-performance liquid chromatography (HPLC) fractionation of the digest.

*Synthesis of compounds containing the cyclopropane ring*

1-(4-chlorophenyl sulphonyl)-1-cyclopropane carbonitrile was prepared from 4-chlorophenyl sulphonyl acetonitrile (Beck and Gunther, 1973) and dibromoethane following published procedures (Takahasi *et al.*, 1985). 3-Cyclopropylindeno [1,2-*c*] pyrazol-4-(O-methyl)-oxime was prepared by a three-stage synthesis as follows: 2-cyclopropylcarbonyl-1,3-indanedione was prepared from dimethyl phthalate and methyl cyclopropyl ketone (Kilgore *et al.*, 1945). The resulting 2-cyclopropylcarbonyl-1,3-indanedione was then reacted with hydrazine hydrate in methanol to yield 3-cyclopropylindeno [1,2-*c*]pyrazole-4-one (Lemke *et al.*, 1982). This was then reacted with O-methyl hydroxylamine hydrochloride in pyridine to yield 3-cyclopropylindeno [1,2-*c*] pyrazol-4-(O-methyl)oxime. The structures of these compounds are given in Figure 1.

**Figure 1** Structures of (**a**) 1-(4-chlorophenyl sulphonyl)-1-cyclopropane carbonitrile; (**b**) 3-cyclopropylindeno [1,2-*c*] pyrazol-4-(O-methyl)oxime and (**c**) ptaquiloside.

*Bracken extracts and spores*

Bracken samples were collected at Benllech, Anglesey, Gwynedd (UK) and extracts were prepared following the method used for the extraction of sesquiterpenoids from *Hypolepis punctata* (Hayashi *et al.*, 1977). Briefly, 1.5 kg of fresh cut bracken was homogenised, extracted in 4 l water and absorbed onto activated charcoal. The absorbed material was eluted with methanol, vacuum dried and reconstituted in water. The spores were collected by Dr J Digby at a site at the University of York, UK.

*Treatment of animals*

Groups of six 8-week-old female $BDF_1$ mice (C57B1 × $DBA_2$; 25–30 g body weight maintained on normal laboratory diet) were given, by gavage under light fluorothane anaesthesia, either 0.25 ml of water as a control, 25 mg bracken spores per mouse (0.25 ml of 100 mg $ml^{-1}$ sonicate in saline) or 0.25 ml of an aqueous extract of bracken frond. The dose of bracken extract was calculated to contain approximately 8 mg ptaquiloside based on a published bracken fern content (Hirono, 1986). The compounds, 1-(4-chlorophenyl sulphonyl)-1-cyclopropane carbonitrile and 3-cyclopropylindeno [1,2-*c*] purazol-4-(O-methyl)oxime, were administered at a dose of 375 mg $kg^{-1}$ in corn oil, with a control group of animals treated with corn oil. Animals were sacrificed at 5 and 24 h after treatment. Liver and the entire upper gastrointestinal tract (stomach and small intestine) were taken, the gut contents were removed and the tissue rinsed in saline. Tissue samples from the animals in the same treatment group were frozen at approximately 70°C and pooled for DNA extraction.

*DNA adduct analysis*

DNA was isolated from the upper gastrointestinal tract and liver by standard phenol-based techniques and 25 $\mu$g DNA was digested to nucleotides using methods described previously (Haque *et al.*, 1994; Povey and Cooper 1995) in 10 mM Tris-HCl containing 5 mM calcium chloride (pH 7.4) using MN (0.5 units $\mu g^{-1}$ DNA) and CSPDE (0.4 m units $\mu g^{-1}$ DNA) overnight at 37°C in a total volume of 62.5 $\mu$l. Aliquots of this digest (between 4 and 16 $\mu$g) were then extracted with butanol (Gupta, 1985), neutralised with 1 $\mu$l of 0.5 M Tris-HCl (pH 9.5) and dried *in vacuo*. In some experiments 2 pmol dIp was added to act as an internal labelling standard.

The adducted nucleotides were $^{32}P$-post-labelled for 1 h at 37°C in 30 mM Tris-HCl (pH 9.5) containing 10 mM magnesium chloride, 10 mM DTT, 1 mM spermidine, 2.9 $\mu$M ATP (total concentration) using 2.5 units $T_4$ PNK and 40 $\mu$Ci $^{32}P$-$\gamma$-ATP in a total volume of 10 $\mu$l. To separate adducted nucleotides, 5 $\mu$l of the labelling mix was chromatographed on plastic-PEI cellulose plates using as dimensions: D1, 1.0 M sodium phosphate (pH 6.5); D3, 3.5 M lithium formate, 8.5 M urea (pH 3.5); D4, 1.2 M lithium chloride, 0.5 M Tris, 8.5 M urea (pH 8) and D5, 1.7 M sodium phosphate (pH 6.0). To ensure that the detected adducts ran into the middle of the thin layer chromatography (TLC)

sheet, each plate was run twice with D3 and D4. When the internal standard was added, 1 μl aliquots of the labelling reaction were chromatographed on glass PEI-cellulose plates (Schleicher-Schuell) using 1 M ammonium formate (pH 3.5) to separate the internal standard from the other radioactive compounds present.

**Figure 2** Phosphorimages of DNA adducts detectable in the gastrointestinal tract of mice following treatment with bracken extract (**a** and **b**), bracken spores (**c** and **d**) or vehicle control (**e** and **f**) at 5 h (**a**, **c** and **e**) and 24 h (**b**, **d** and **f**). Levels of adducts marked 1, 2 and 3 are quantified in Table I.

**Table I** Level of adducts induced by bracken extract and bracken spores present in gastrointestinal tissue DNA

| *Sample* | *Time (h)* | *Adduct level (nmol mol$^{-1}$ dGuo)$^a$* 1$^b$ | 2 | 3 |
|---|---|---|---|---|
| Bracken extract | 5 | 2.0 ± 0.6 | 6.3 (9.5 ± 1.2)$^c$ | 3.9 ± 0.6 (3.3 ± 2.0) |
| | 24 | <0.1 | 0.8 ± 0.3 (<0.1) | 0.9 ± 1.3 (<0.1) |
| Bracken spores | 5 | 1.1 ± 0.6 | 6.6 ± 1.6 (10.9 ± 3.6) | 3.8 ± 1.6 (3.0 ± 0.5) |
| | 24 | 1.5 ± 0.6 | 8.0 ± 1.3 (7.0 ± 0.5) | 2.2 ± 2.1 (4.9 ± 0.6) |
| Vehicle control | 5 | <0.1 | 0.1 ± 0.2 (<0.1) | 0.2 ± 0.3 (<0.1) |
| | 24 | <0.1 | <0.1 | <0.1 |

[a]Results are given as mean ± s.d. ($n = 3$). [b]The TLC position of adducts 1, 2 and 3 are shown in Figure 2. [c]Results in brackets are for those determined using an internal standard method for determining adduct levels.

*Quantitation of normal nucleotides*

To determine normal levels of nucleotides released, 10 $\mu g$ aliquots of the same DNA digest were treated with alkaline phosphatase to give nucleotides that were then quantified by HPLC (Cooper *et al.*, 1992).

*Adduct quantitation*

TLC plates were dried, wrapped in plastic wrap and exposed to a phosphor screen (Molecular Dynamics, Sunnyvale, CA, USA) for up to 48 h. Sample visualisation was carried out using a Molecular Dynamics 425S phosphorimager at 176 $\mu m$ resolution and volume quantitation was carried out using Imagequant software (Molecular Dynamics). Background determination for each individual image was carried out by calculating the average of all pixel values in the outline of the object.

In the absence of an internal standard, adducts were quantified by comparing the signal with that of known amounts of $^{32}P$ spotted onto the same TLC plates and exposed together on the same phosphor screen for up to 48 h. The amounts of adducts detected were then quantified by determining the (adduct signal / $^{32}P$ signal) ratio and assuming that the adduct was labelled quantitatively. When the internal standard was added, it was found that the response of the phosphor screens to the signal from the internal standard was linear only for a relatively short period of time (<5 h). It was thus not possible to compare directly the signal from the internal standard with that from the adduct over an extended period (48 h). A standard curve, for which the phosphor screen response was linear for up to 48 h, was then prepared by diluting stock [$^{32}P$]-$\gamma$-ATP between 100 and $10^6$ times. The same standard curve was then exposed to each 1D plate (containing the internal standard) and each Randerath plate (containing the adducts) for 2 and 48 h respectively. Hence, the total amount of adduct present was equal to (adduct signal)/(standard signal 48 h) $\times$ (standard signal 2 h)/pdIp signal) $\times$ 400 fmol (to account for differences in the amounts spotted on the different plates). In the absence of chemically synthesised adduct standards, we have assumed that the adduct was labelled at the same efficiency as the internal standard. Adduct levels are expressed as nmol adducts detected per mol of dGuo released (as determined by HPLC).

### Results

A cluster of adducts, not present in the vehicle control, was clearly seen in the analyses of the butanol extracts from the digests of DNA from the upper gastrointestinal tract obtained 5 and 24 h after treatment with bracken extract (Figure 2). Furthermore, a similar adduct pattern was observed in the DNA of those animals treated with bracken spores. Although there appeared to be a number of minor DNA adducts, three adducts (1–3; Figure 2a) were consistently observed and therefore quantified (Table I). After 5 h, adduct levels in extract- and spore-treated animals were similar (mean levels ranging from 1.1 to 6.6 nmol $mol^{-1}$ dGuo) but, whereas after 24 h adduct levels in the extract-treated animals had diminished by >75%, levels in spore-treated animals were similar to those found after 5 h. This suggests that the DNA reactive compound(s) was being released slowly from the spores, even though the spores had been sonicated before administration. Liver DNA from treated mice did not exhibit the same pattern of adducts, but only adducts that were detected in both control and treated animals (data not shown).

The levels of bracken-induced adducts were also quantified after the addition of an internal standard (dIp) to account for variations in labelling efficiency. Figure 3 clearly demonstrates that pdIp could be separated simply by one-dimensional chromatography from contaminating nucleotides and residual ATP. We found that quantitative labelling of dIp in the butanol extract could be obtained only when

**Figure 3** Phosphorimages of $^{32}P$-labelled samples run one dimensionally with (**a**) or without (**b**) the internal standard (dIp) present. Lane 1, dIp; lane 2, a water blank and lanes 3–5, butanol-extracted adducts arising from bracken. Positions of [$^{32}P$]-$\gamma$-ATP, pdIp and pdNp are marked on the images.

samples of <4 $\mu g$ DNA were digested and extracted. With increasing amounts of DNA, higher levels of residual nucleotides resulted in suboptimal labelling of dIp. Results for the quantitation of adduct levels using the internal standard approach were similar to those found in the absence of the dIp, indicating that quantitative labelling of the adducts had occurred in both the presence and absence of the standard. There appeared to be no differences in the variation of results obtained.

As part of this work, we have prepared a number of compounds on the route to a synthesis of ptaquiloside. This synthesis has so far been unsuccessful owing to the instability of the cyclopropane ring during the multistep synthesis. However, treatment of mice with 1-(4-chlorophenyl sulphonyl)-1-cyclopropane carbonitrile and 3-cyclopropylindeno [1,2-*c*]pyrazol-4-(O-methyl)oxime resulted in the formation of adducts that were detectable in the DNA of the upper gastrointestinal tract (Figure 4). The adduct pattern was similar to those seen with bracken spores and bracken extract: preliminary experiments indicated that these adducts co-chromatographed with those adducts derived from bracken spores or extract using the same urea-based solvent systems or when using ammoniumn hydroxide/isopropanol as D4 (Beach and Gupta, 1992).

### Discussion

In this study we have demonstrated that DNA adducts arising from exposure to bracken extract or bracken spores can be detected in the upper gastrointestinal tract of mice by $^{32}$P-post-labelling. As ptaquiloside is thought to be the principal carcinogenic component in bracken (Hirono *et al.*, 1987) and reacts directly with nucleotides *in vitro* (Ojika *et al.*, 1987), these DNA adducts, detected by $^{32}$P-post-labelling, may result from the interaction of an unstable dienone, formed by the breakdown of ptaquiloside, with DNA. Their identity is as yet unknown, but as $T_4$-PNK only phosphorylates adducts with a bulky substituent at the N7 position of guanine poorly (Koivisto and Hemminki, 1990), an adduct at the 7-guanine position would probably not have been detected. The N3 adenine and N7 guanine ptaquiloside adducts identified previously (Kushida *et al.*, 1994) spontaneously depurinate *in vitro* with $t_{1/2}$ of 18 and 40 h respectively. As more than 75% of the adduct levels present at 5 h, in mice treated with bracken extract, had been removed by 24 h, the adducts detected in this study were probably not these N3 and N7 modifications, unless these

**Figure 4** Phosphorimages of DNA adducts detectable in the gastrointestinal tract of mice following treatment with 1-(4-chlorophenyl sulphonyl)-1-cyclopropane carbonitrile (**a** and **b**) and 3-cyclopropylindeno [1,2-*c*] pyrazol-4-(O-methyl) oxime (**c** and **d**) or vehicle control (**e** and **f**) at 5 h (**a**, **c** and **e**) and 24 h (**b**, **d** and **f**).

lesions are removed by active repair processes as is the case with N3 and N7 methylguanine adducts (Saffhill *et al.*, 1985).

In order to generate the appropriate marker compounds and so to provide definitive proof that these adducts arise from ptaquiloside, we have tried to synthesise ptaquiloside itself, but this has proved difficult owing to the instability of the cyclopropyl ring (Ojika *et al.*, 1987). Two other compounds containing a cyclopropyl ring, namely 1-(4-chlorophenyl sulphonyl)-1-cyclopropane carbonitrile and 3-cyclopropylindeno [1,2-*c*]pyrazol-4-(O-methyl)oxime, were synthesized and, when administered to mice, formed DNA adducts in gastrointestinal tissue. The pattern of these adducts was similar to that obtained with bracken extract and bracken spores and initial co-chromatography experiments indicated that adducts arising from these three compounds could not be resolved. These results suggest that adducts formed in mice treated with bracken extract or spores may have arisen from ptaquiloside, as this compound, as well as 1-(4-chlorophenyl sulphonyl)-1-cyclopropane carbonitrile and 3-cyclopropylindeno [1,2-*c*]pyrazol-4-(O-methyl)oxime, contains a cyclopropyl ring. However, whether these adducts are in fact derived from the compounds and bracken spores/extract *per se* or arise via another mechanism, e.g. increased formation of endogenous adducts such as I compounds (Randerath *et al.*, 1986), remains to be determined. One possible approach to identify these adducts would be to synthesise ptaquiloside-DNA adducts chemically so as to determine whether these adducts are substrates for $T_4$-PNK and can be detected using our assay and chromatographic conditions.

Bracken consumption is associated with increased cancer risk in Japan (Hirayama, 1979) and Brazil (Marliere *et al.*, 1995). Within the UK, exposure is now almost certainly confined to bracken spores at times when bracken is sporulating. At a site near Caernarfon in North Wales (UK), the bracken spore count in a bracken stand rose from zero levels over the course of 4–5 days to counts of $>800$ per l or $>9600$ per l $h^{-1}$ and then fell to low levels. These values equate to approximately 480 000 spores per hour in the respiratory tidal flow volume. For comparison, when air was sampled in a control, non-bracken area, the count remained low (Heyworth *et al.*, unpublished data; Povey *et al.*, 1995). As part of these exploratory studies, it was also shown that walking for 15 min in a stand of sporulating bracken while wearing a face mask, resulted in deposition on the surface of the filter at the rate of approximately 11 mg of spores $h^{-1}$. As we have demonstrated that DNA reactive compounds can be released from spores, inhalation of bracken spores may thus provide a significant route of exposure.

In this study, we have also used a modified version of the assay (Shields *et al.*, 1993) in which an internal standard is added to the isolated DNA adducts in order to minimise variations in labelling efficiency and have found that bracken-induced adduct levels were similar whether or not an internal standard was added (Table I). This is in contrast to previous results in which it was reported that the addition of an internal standard reduced the sensitivity of the assay to detect polycyclic aromatic hydrocarbon adducts (Shields *et al.*, 1993). This suggests that the bracken-induced adducts are labelled quantitatively in both procedures: the absolute amount of adducts detected, however, remains to be determined, as adduct losses may occur during the DNA extraction, digestion and butanol extraction steps. Rather than measuring the amount of internal standard directly as described previously (on the PEI-cellulose plate before removal of the excess [$^{32}$P]-$\gamma$-ATP; Shields *et al.*, 1993), we have found it simpler to chromatograph a separate aliquot which enables the identification of both [$^{32}$P]-$\gamma$-ATP and the internal standard [$^{32}$P]pdIp (see Figure 3). Although the actual procedure for quantitation is more complex than the direct measurement of radioactivity incorporated, potentially this approach can allow for variations in labelling efficiency. As we add a known amount of an internal standard into the labelling reaction, we can also directly determine whether the labelling reaction had, in fact, occurred to the extent expected. For example, although we have found that we can detect adducts after extraction of digests of $>10$ $\mu$g DNA, the reproducibility of adduct quantitation was poor owing to increased levels of contaminating normal nucleotides that had been coextracted. Under these conditions, labelling of the internal standard was also reduced. This was not a problem, however, with DNA digests containing $<4$ $\mu$g DNA. These observations suggest that addition of an internal standard may aid in increasing the reliability and reproducibility in adduct measurement, even for aromatic adducts.

In summary, we have now shown for the first time that DNA adducts can be detected in rodents treated with either bracken extract or bracken spores using a $^{32}$P-post-labelling procedure commonly employed to investigate DNA damage in human populations. As ptaquiloside is a known DNA-damaging agent and is the principal carcinogenic component in bracken, these adducts may arise from the interaction of ptaquiloside with DNA. However, a comparable adduct pattern was observed using two other compounds, whose structures were only similar to ptaquiloside, in that they contained a cyclopropyl ring, suggesting that these compounds and bracken spores/extract may have induced the formation of adducts by an indirect mechanism. As humans are exposed to a large number of potential carcinogens, further work will be necessary to develop methods that are specific for the isolation of bracken-induced DNA adducts, e.g. immunocolumns (Cooper *et al.*, 1992), before human exposure can be definitively demonstrated and accurately quantified.

### Abbreviations

MN, micrococcal nuclease; CSPDE, calf spleen phosphodiesterase; dIp, deoxyinosine 3′-monophosphate; pdIp, deoxyinosine 3′,5′-bisphosphate; pdNp, 3′,5′-bisphosphates of normal nucleosides; $T_4$-PNK, $T_4$-polynucleotide kinase.

### Acknowledgements

The authors are grateful for support for this project from the Gunnar Nilsson Cancer Research Trust fund, the Oakdale Trust and the William A Cadbury Charitable Trust and to the Cancer Research Campaign for long-term funding. We also wish to thank Professor JA Taylor for his continuing interest.

### References

BEACH AC AND GUPTA RC. (1992). Human biomonitoring and the $^{32}$P-postlabelling assay. *Carcinogenesis*, **13**, 1053–1074.

BECK G AND GUNTHER D. (1973). A new synthesis of 1,2,3-triazoles. *Chem. Ber.*, **106**, 2758.

COOPER DP, GRIFFIN KA AND POVEY AC. (1992). Immunoaffinity purification combined with $^{32}$P-postlabelling for the detection of $O^6$-methylguanine in DNA from human tissue. *Carcinogenesis*, **13**, 469–475.

EVANS IA. (1984). Bracken carcinogenicity. In *Chemical Carginogens*, Volume 2. Searle CE (ed.) pp. 1171–1204. American Chemical Society: Washington DC.

EVANS IA. (1987). Bracken carcinogenicity. In *Reviews on Environmental Health. Int. Quart. Sci. Reviews*, Vols 3/4. James RV. (ed.) pp. 161–169. Freund Publishing House Ltd: Tel Aviv, Israel.

EVANS IA AND MASON J. (1965). Carcinogenic activity of bracken. *Nature*, **208**, 913–914.

GALPIN OP, WHITAKER CJ, WHITAKER RL AND KASSAAB JY. (1990). Gastric cancer in Gwynedd: possible links with bracken. *B. J. Cancer*, **61**, 737–740.

GUPTA RC. (1985). Enhanced sensitivity of $^{32}$P-postlabelling analysis of aromatic: carcinogen DNA adducts. *Cancer Res.*, **45**, 5656–5662.

HAQUE K, COOPER DP AND POVEY AC. (1994). Optimisation of $^{32}$P-postlabelling assays for the quantitation of $O^6$-methyl and N7-methyldeoxyguanosine 3′-monophosphates in human DNA. *Carcinogenesis*, **15**, 2485–2490.

HAYASHI Y, NISHIZAWA M AND SAKAN T. (1977). Studies on the sesquiterpenoids of *Hypolepis punctata* Mett: isolation and structure determination of hypacrone, a new seco-illudiod. *Tetrahedron*, **33**, 2509–2511.

HIRAYAMA T. (1979). Diet and cancer. *Nutr. Cancer*, **1**, 67–81.

IARC. (1986). Bracken fern. In *Monographs on the Evaluation of Carcinogenic Risk to Humans No. 40. Some Naturally Occuring and Synthetic Food Components, Fourocoumarins and Ultraviolet Radiation*. pp. 47–65. WHO, IARC: Lyon.

HIRONO I. (1986). Carcinogenicity of plant constituents: pyrrolizidine alkaloids, bracken fern. In *Genetic Toxicology of the Diet*, Knudsen I. (ed.) pp. 45–53. Alan Liss Inc: New York.

HIRONO I, OGINO H, FUJIMOTO M, YAMADA K, YOSHIDA Y, IGAWA M AND OKUMURA M. (1987). Induction of tumours in ACI rats given a diet containing ptaquiloside, a bracken carcinogen. *J. Natl Cancer Inst.*, **79**, 1143–1149.

KILGORE LB, FORD JH AND WOLFE WC. (1945). Insecticidal properties of 1,3-indanediones. *Ind. Eng. Chem.*, **34**, 494–497.

KOIVISTO P AND HEMMINKI K. (1990). $^{32}$P-postlabelling of 2-hydroxyethylated, ethylated and methylated adducts of 2′-deoxyguanosine 3′-monophosphate. *Carcinogenesis*, **11**, 1389–1392.

KUSHIDA T, UESUGI M, SUGIURA Y, KIGOSHI H, TANAKA H, HIROKAWA J, OJIKA M AND YAMADA K. (1994). DNA damage by ptaquiloside, a potent bracken carcinogen: detection of selective strand breaks and identification of DNA cleavage products. *J. Am. Chem. Soc.*, **116**, 479–486.

LEMKE TL, SAWKNEY KN AND LEMKE BK. (1982). Synthesis and chemical reactivity of indenoisoxazoles. *J. Heterocyclic Chem.*, **19**, 363–368.

LENFESTEY JH. (1972/73). The Guernsey 'green bed'. In *The Channel Islands Annual Anthology*. No. 1. Stevens-Cox J and Stevens-Cox G (eds). pp. 69–91. Toucan Press: St Peter Port, Channel Islands.

MARLIERE CA, GALVAO MAM, SANTOS RC, KAWAMOTO M, SILVA MLC, BARRETTO JMA AND GOMES RQF. (1995). Gastric and oesophageal cancer: possible links with bracken (*Pteridium aquilinum*) ingestion – a case–control study in Ouro Preto MG Brazil. In *Bracken: an Environmental Issue*. Smith RJ and Taylor JA (eds). pp. 99–101. University of Leeds: Leeds.

OJIKA M, WAKAMATSU K, NIWA H AND YAMADA K. (1987). Ptaquiloside, a potent carcinogen isolated from bracken fern *Pteridium aquilinum var latiusculum:* structure elucidation based on chemical and spectral evidence and reactions with amino acids, nucleosides and nucleotides. *Tetrahedron*, **43**, 5261–5274.

OJIKA M, SUGIMOTO K, OKAZAKI T AND YAMADA K. (1989). Modification and cleavage of DNA by ptaquiloside. A new potent carcinogen isolated from bracken fern. *J. Chem. Soc. Chem. Commun.*, **22**, 1775–1777.

POVEY AC AND COOPER DP. (1995). The development, validation and application of a $^{32}$P-postlabelling assay to quantify $O^6$-methylguanine in human DNA. *Carcinogenesis*, **16**, 1665–1669.

POVEY AC, EVANS IA, TAYLOR JA AND O'CONNOR PJ. (1995). Detection of DNA adducts by $^{32}$P-postlabelling in rats treated with bracken extract and bracken spores. In *Bracken: an Environmental Issue*. Smith RJ and Taylor JA (eds). pp. 95–98. University of Leeds: Leeds.

RANDERATH K, REDDY MV AND DISHER RM. (1986). Age- and tissue-related DNA modifications in untreated rats: detection by $^{32}$P-postlabelling assay and possible significance for spontaneous tumour induction and aging. *Carcinogenesis*, **7**, 1615–1617.

SAFFHILL R, MARGISON GP AND O'CONNOR PJ. (1985). Mechanisms of carcinogenesis induced by alkylating agents. *Biochim. Biophys. Acta*, **823**, 111–146.

SHIELDS PG, HARRIS CC, PETRUZZELLI S, BOWMAN ED AND WESTON A. (1993). Standardization of the $^{32}$P-postlabelling assay for polycyclic aromatic hydrocarbon-DNA adducts. *Mutagenesis*, **8**, 121–126.

SMITH BL AND SEAWRIGHT AA. (1995). Bracken fern (*Pteridium* spp.). Carcinogenicity and human health – a brief review. *Natural Toxins*, **3**, 1–5.

TAKAHASI M, SUZUKI H AND KATA Y. (1985). Synthesis of 1–phenylsulfonylcyclopropanecarboxylic acid derivatives. *Bull. Chem. Soc. Jpn.*, **58**, 765–766.

TAYLOR JA. (1986). The bracken problem: a local hazard and global issue. In *Bracken: Ecology, Land Use and Control Technology*, Smith RT and Taylor JA. (eds). pp. 21–42. Parthenon Publishing: Carnforth UK.

VILLALOBOS-SALAZAR J, MENESES A, ROJAS JL, MORA J, PORRAS RE AND HERRERO MV. (1989). Bracken derived carcinogens as affecting animal and human health in Costa Rica. In *Bracken Toxicity and Carcinogenicity as related to Human Health*. Taylor JA (ed.). pp. 40–45. International Bracken Group: Aberystwyth.

VILLALOBOS-SALAZAR J, MENESES A AND SALAS J. (1990). Carcinogenic affects in mice of milk from cows fed on bracken from *Pteridium aqulinum*. In *Bracken Biology and Management*. Thomson JA and Smith RA. (eds). pp. 247–251, OCCAS Publication no. 40. Australian Institute of Agricultural Science: NSW.